# SAMHD1 transcript upregulation during SIV infection of the central nervous system does not associate with reduced viral load

**DOI:** 10.1038/srep22629

**Published:** 2016-03-03

**Authors:** Erin L. Buchanan, Diego A. Espinoza, Melissa A. McAlexander, Stephanie L. Myers, Adam Moyer, Kenneth W. Witwer

**Affiliations:** 1 Department of Molecular and Comparative Pathobiology, 733 North Broadway, Baltimore, Maryland, 21025, USA; 2 Cellular and Molecular Medicine Graduate Program, 733 North Broadway, Baltimore, Maryland, 21025, USA; 3 Department of Neurology, The Johns Hopkins University School of Medicine, 733 North Broadway, Baltimore, Maryland, 21025, USA

## Abstract

Restriction of HIV-1 in myeloid-lineage cells is attributed in part to the nucleotidase activity of the SAM-domain and HD-domain containing protein (SAMHD1), which depletes free nucleotides, blocking reverse transcription. In the same cells, the Vpx protein of HIV-2 and most SIVs counteracts SAMHD1. Both Type I and II interferons may stimulate SAMHD1 transcription. The contributions of SAMHD1 to retroviral restriction in the central nervous system (CNS) have been the subject of limited study. We hypothesized that SAMHD1 would respond to interferon in the SIV-infected CNS but would not control virus due to SIV Vpx. Accordingly, we investigated SAMHD1 transcript abundance and association with the Type I interferon response in an SIV model. SAMHD1 transcript levels were IFN responsive, increasing during acute phase infection and decreasing during a more quiescent phase, but generally remaining elevated at all post-infection time points. *In vitro*, SAMHD1 transcript was abundant in macaque astrocytes and further induced by Type I interferon, while IFN produced a weaker response in the more permissive environment of the macrophage. We cannot rule out a contribution of SAMHD1 to retroviral restriction in relatively non-permissive CNS cell types. We encourage additional research in this area, particularly in the context of HIV-1 infection.

The sterile alpha motif and histidine-aspartate domain-containing protein 1 (SAMHD1) is a 72-kD protein originally characterized as the human ortholog^1^ of a mouse gamma interferon-induced gene^2^. SAMHD1 was later found to have mutations associated with Aicardi-Goutières syndrome^3^. More recently, SAMHD1 was identified as a host restriction factor for HIV-1^4^,^5^. Restriction factors block or interfere with one or more stages of the viral life cycles and include but are not limited to members of the APOBEC cytidine deaminase family, tetherin, TRIM5alpha, and cellular microRNAs^6^,^7^,^8^.

SAMHD1 in its activated state depletes the cellular pool of deoxynucleotides through deoxynucleoside triphosphohydrolase activity^9^,^10^,^11^. Depleting the dNTPs needed to build genomic DNA, SAMHD1 thus exerts antiviral restriction by slowing or stopping the reverse transcription step of HIV-1 replication. Consistent with the antiviral role of SAMHD1, cells from individuals with Aicardi-Goutières syndrome have heightened susceptibility to HIV-1 infection^12^. Exonuclease activity of SAMHD1 has also been described^13^. The Vpx accessory proteins of HIV-2 and SIV target SAMHD1 for interaction with the E3 ubiquitin ligase complex^14^, resulting in proteasome-mediated degradation and counteraction of the restriction activity^4^,^5^,^15^. The Vpr protein of some primate lentiviruses can also orchestrate SAMHD1 degradation^16^.

SAMHD1 expression and its control have been examined extensively in certain cell types. SAMHD1 has been found to be relatively highly expressed in cells that are restrictively permissive to HIV infection, such as macrophages and dendritic cells of myeloid cell origin^17^ or resting CD4+ T-cells^18^. In contrast, some permissive cells may display lower levels of SAMHD1. Transcription of SAMHD1 can be stimulated by interferon gamma^2^,^19^. There is also evidence for stimulation by Type I interferons^12^,^20^,^21^,^22^,^23^, but evidence against interferon stimulation has also been presented^20^,^24^,^25^. At the same time, in some cells, SAMHD1 may be part of a feedback system, as it has been reported to antagonize the expression of certain Type I IFN-stimulated genes^26^,^27^, strikingly in monocyte-derived dendritic cells^28^. However, some myeloid-lineage cells and resting CD4+ T-cells from individuals with SAMHD1-inactivating mutations supported robust infection *in vitro*^12^,^29^, suggesting that the observed anti-IFN activity of SAMHD1 is cell type specific or that its ablation is insufficient to allow the interferon response to protect against HIV-1 infection. Differential transcript-level expression of SAMHD1 has been reported in cells of one cohort of HIV-1 elite suppressors^30^, but not in two others^31^,^32^.

In contrast with the periphery, relatively little is known about SAMHD1 levels during retroviral infection of the brain and progression to the HIV-associated neurocognitive disorders (HAND). Neurologic correlates of HIV infection have been observed since the earliest days of the pandemic^33^,^34^,^35^ and may even occur despite successful virologic control^36^,^37^,^38^. Some pharmacologic agents may contribute to HAND^39^ by inducing oxidative stress. Infiltrating macrophages and resident microglia are productively infected^40^. Astrocytes, the most abundant cells in the brain, are also infected^41^, albeit rarely productively *in vivo*. In one study of SAMHD1 in CNS cells, interferon alpha transiently increased SAMHD1 production in immortalized human stem cell-derived astrocytes^23^. In another, astrocytes were found to have higher levels of SAMHD1 than microglia, which are relatively permissive to infection^42^. Host miR-181a was found to regulate SAMHD1 *in vitro*^42^, although miRNA association with SAMHD1 levels was not apparent *in vivo*^43^.

Since SAMHD1 transcription and Type I interferon responsiveness in the retrovirus-infected CNS are unknown, we hypothesized that SAMHD1 transcript levels might respond to SIV infection, but without affecting viral replication due to the action of SIV Vpx. We further hypothesized that macaque astrocytes, which are relatively refractory to infection and host only low levels of viral replication, would display high levels of SAMHD1 transcription (as now also reported for human astrocytes^42^). We therefore examined transcription of SAMHD1 in thalamus in an SIV model^44^ in which an acute infection phase of ten to 14 days includes peak cytokine responses^45^ and is followed by a latent or asymptomatic phase. Recapitulation of markers of human disease progression is accompanied by AIDS and CNS disease by three months post-infection in >90% of cases, with sustained inflammation and other factors contributing to neurodegenerative pathologies^46^. SAMHD1 transcript was also measured in primary macaque astrocytes infected with SIV and/or treated with interferon beta, the Type I interferon most important in the CNS response to viral replication.

Our results indicate that SAMHD1 mRNA expression in thalamus mirrors the rise of proinflammatory cytokine expression during acute infection and appears to remain elevated during asymptomatic infection and into disease progression. *In vitro*, primary macrophages and astrocytes did not undergo significant changes in SAMHD1 expression during the course of long-term infection with HIV or SIV, although early changes were observed in SIV-infected astrocytes. However, treatment of macrophages and especially astrocytes with exogenous interferon-beta resulted in significant fold changes in SAMHD1.

## Results

### Regulation of SAMHD1 transcript in CNS during retroviral infection

**Figure 1:**
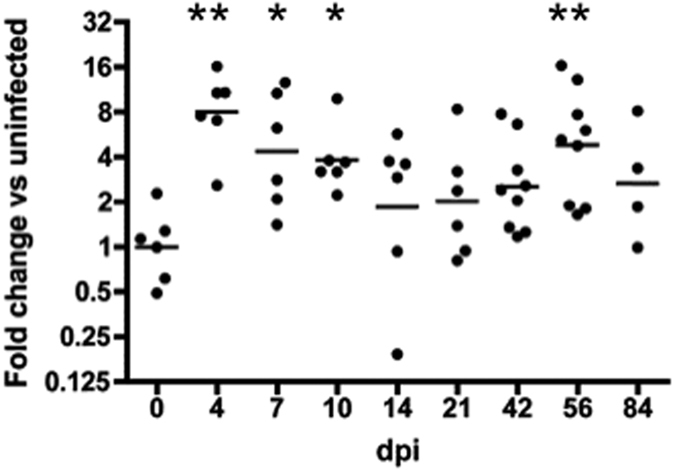
Brain SAMHD1 transcript load during SIV infection. SAMHD1 transcript increased in thalamus during SIV infection, with peak expression corresponding with acute and late-stage infection. SAMHD1 mRNA was quantitated by qPCR, with normalization to the average of beta actin and GAPDH. **p < 0.01, *p < 0.05.

Thalamus RNA samples from 58 subjects (Table 1) were obtained and subjected to quantitative PCR. With reference to the average of two housekeeping genes, GAPDH and beta actin, and compared with levels in six uninfected control animals, SAMHD1 was significantly increased during acute infection (4, 7, and 10 dpi) (Fig. 1). Indeed, an increase over baseline was maintained at later time points in all but a small number of subjects, even during the “latent” or “asymptomatic” phase that includes 14 and 21 dpi. This increase was nominally significant at 56 dpi, but this should be viewed in the context of the broader trend towards increased expression.

### SAMHD1 and the Type I interferon response

**Figure 2:**
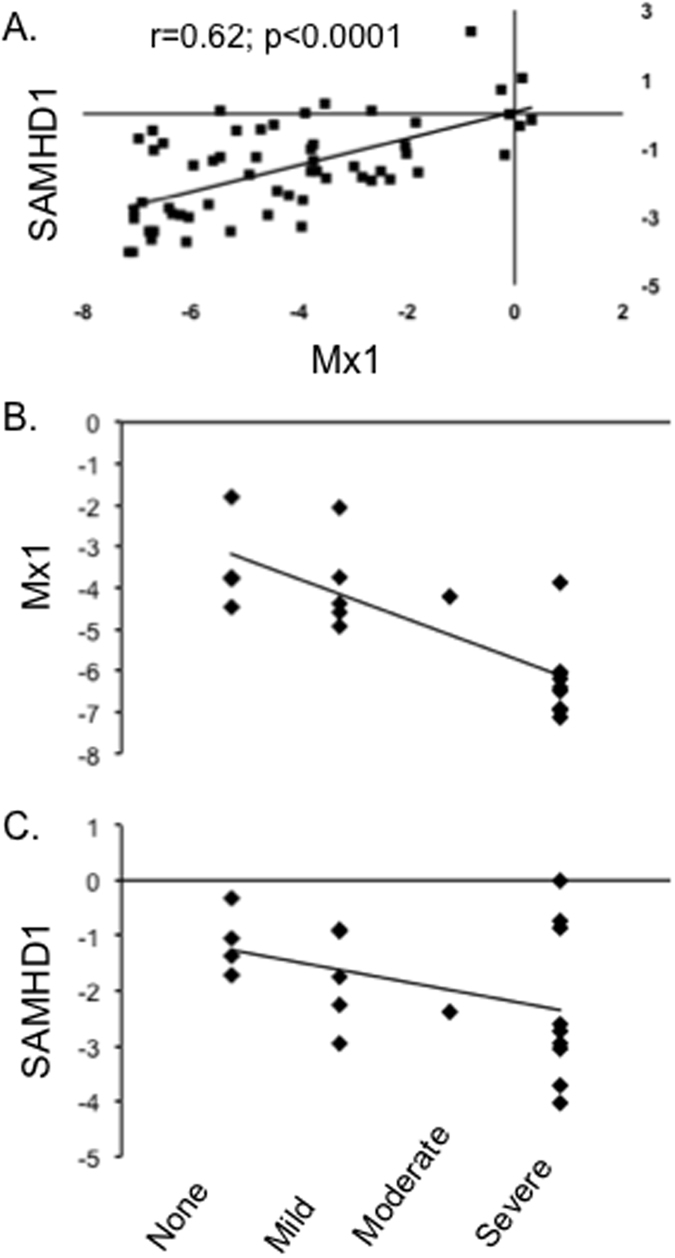
Relationship of SAMHD1 transcription, Type I interferon response, and disease severity. There was a significant positive correlation of SAMHD1 transcript and level of MX1, an indicator of the Type I interferon response (**A**). Comparing Mx1 (**B**) and SAMHD1 (**C**) transcript with disease severity as assigned by pathology (none, mild, moderate, or severe), the association of Mx1 with disease appeared stronger than the association of SAMHD1 and disease. Transcript abundance was normalized to the average of beta actin and GAPDH; hence, the smaller the “dCq” value, the greater the abundance.

Based upon reports in the literature and our experience with measuring host antiviral responses^45^, we postulated that the observed increases in SAMHD1 are part of the Type 1 interferon antiviral program. To investigate this possibility, we first used a bioinformatics approach to confirm that the macaque SAMHD1 promoter sequence, like that of human, contains interferon stimulated response elements (ISREs). MatInspector predicted ISREs in both human and animal sequences (Table 2). Next, we compared expression of SAMHD1 and the interferon stimulated gene Mx1 (Fig. 2A). A strong correlation between the two was observed. This finding is consistent with Type I interferon responsiveness of both genes.

### SAMHD1 transcript during lentiviral brain disease development

**Figure 3:**
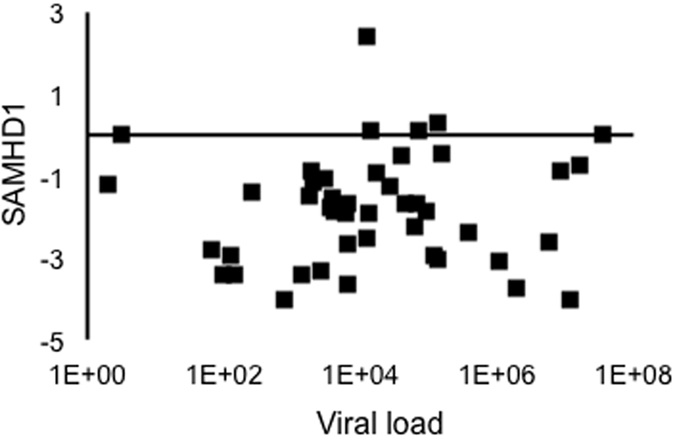
Viral load and SAMHD1. Tissue viral load as determined by standard curve-normalized qPCR did not correlate with SAMHD1 transcript (normalized to the average of beta actin and GAPDH).

In the rapid model, disease develops by the 42 and 56 day post-inoculation time points. While Mx1 was correlated with CNS disease severity (Fig. 2B), SAMHD1 displayed a weaker correlation with disease (Fig. 2C) at these time points. A trend towards greater expression of SAMHD1 in severe disease was partially offset by several “non-responsive” animals with lower SAMHD1 expression despite high MX1 response (Fig. 2C). As we hypothesized, however, higher levels of SAMHD1 expression did not associate overall with lower viral loads in brain tissue (Fig. 3). Both MX1 and SAMHD1 expression were positively correlated with severity of CNS disease, consistent with a runaway immune response that, in acute phase infection, might be protective, but at later stages becomes damaging.

### SAMHD1 regulation in monocyte-derived macrophages

To follow up on the *in vivo* findings consistent with Type I interferon responsiveness of SAMHD1 in brain, we next sought to determine which cell types might be responsible for SAMHD1 regulation in the CNS. We assayed SAMHD1 expression in several relevant cell types *in vitro*, in response to interferon beta (IFNB1) treatment or infection. The first cell type assayed was the human monocyte-derived macrophage. We studied human macrophages because of their relative availability at the time of these experiments. Following infection, monocyte-lineage cells enter the brain within days, seeding CNS viral infection. During progression to disease, perivascular and parenchymal macrophages support productive infection and release damaging proinflammatory cytokines.

**Figure 4:**
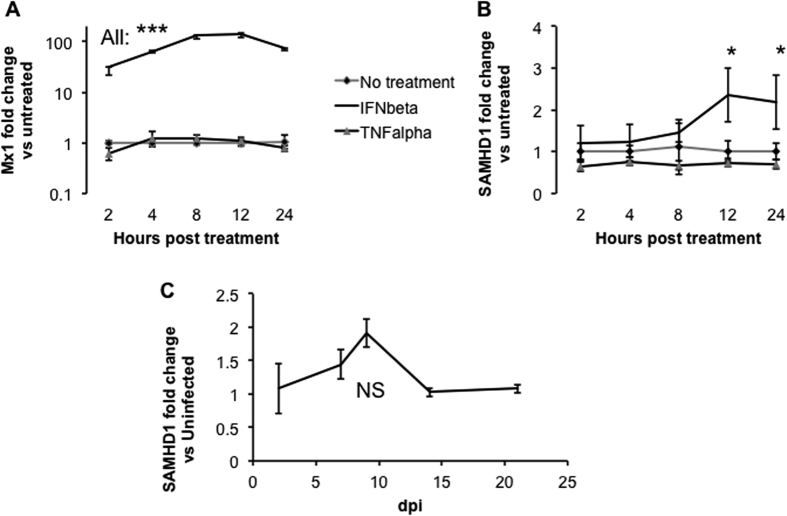
SAMHD1 and IFNB1 in primary macrophages. M×1 of primary human monocyte-derived macrophages responded rapidly and strongly to 100 U/mL treatment with recombinant IFNB1, but not to TNFalpha (20 ng/mL, (**A**)). SAMHD1 also responded to IFNB1, but the increase was detectable and significant only by 12 hours post-treatment (**B**). HIV-1 (BaL) infection of primary monocyte-derived macrophages yielded an apparent increase of SAMHD1 levels by 7 and 10 days post-infection, but this increase was not statistically significant (NS) (**C**). ***p < 0.001, *p < 0.05.

In human monocyte-derived macrophages, differentiated by response to macrophage colony stimulating factor (M-CSF), Mx1 was upregulated rapidly and significantly as early as 2 hours post-treatment with recombinant IFNB1 (Fig. 4A). In contrast, TNFalpha did not trigger significant changes in Mx1. Interferon beta resulted in later upregulation of SAMHD1, beginning between 8 and 12 hours post-treatment (Fig. 4B). This increase was significant at 12 and 24 hours. It is unclear why the effect was delayed for SAMHD1 compared with Mx1. Possibly, the interferon effect is indirect, requiring initiation of a secondary signaling cascade. TNFalpha treatment resulted in apparently lower SAMHD1 expression. However, this trend did not reach significance.

In parallel, we assessed the effect of HIV infection on macrophages. Cells were infected at a multiplicity of infection of 0.05, and samples were harvested at time points including two, seven, nine, 14, and 21 days post-infection. Productive infection was confirmed by p24 ELISA (not shown). Despite an apparent trend towards increased SAMHD1 abundance at seven and nine dpi, none of the changes was significant (Fig. 4C). Given the significant increase in SAMHD1 in interferon beta-treated cells, this result might seem counterintuitive. Would infected or exposed cells not generate interferon and thus render themselves and surrounding cells less susceptible to infection or replication? There are several explanations we can offer. First, macrophage innate responses to HIV infection may be turned off or subverted by viral products or specific cellular responses to viral products^47^. As cited above, according to several reports, SAMHD1 itself can dampen IFN responses under certain circumstances. Second, the timing of the experimental exposure to recombinant interferon may have led to negative feedback by cellular factors such as miRNAs^48^, influencing results. Finally, replicating *in vitro* the microenvironment of the brain during SIV infection may be a considerable challenge.

### SAMHD1 regulation in primary astrocytes

The second cell type we tested was the primary astrocyte. To take advantage of a unique resource—rhesus adult primary astrocytes—this experiment was performed with SIV/17E-Fr infection, not HIV. The most abundant cell type in the brain, astrocytes, are readily infected *in vitro*^49^. They are also infected *in vivo*, but incompletely characterized blocks to replication usually prevent productive infection. By sheer numbers, however, astrocytes comprise the most abundant, if largely inactive, lentiviral reservoir in the CNS.

**Figure 5:**
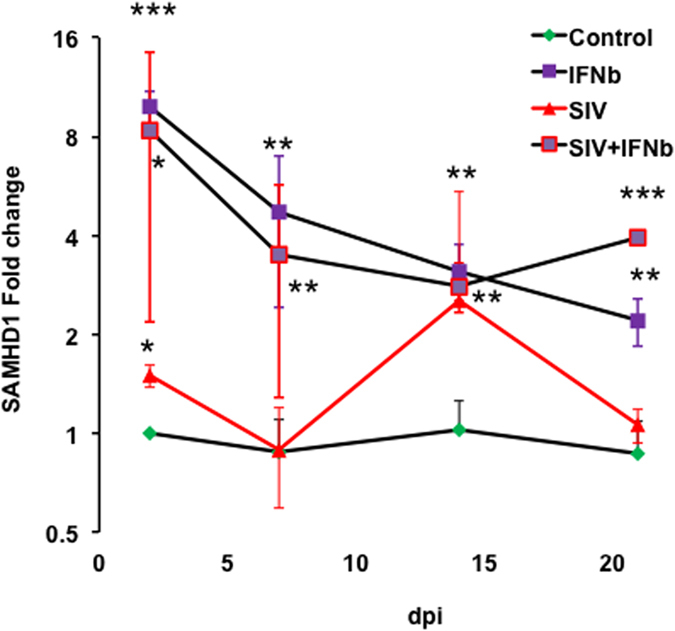
Primary astrocyte SAMHD1 responds to SIV infection and IFNB1. Quantitative PCR results, normalized to the average of beta actin and GAPDH, indicated that SIV infection (SIV 17E-Fr) and IFNB1 treatment of primary rhesus astrocytes resulted in significant upregulation of SAMHD1 by 48 hours post infection and at all subsequent time points examined. SIV infection alone stimulated apparently significant upregulation at two and 14 days post infection. ***p < 0.001, **p < 0.01, *p < 0.05.

Astrocytes infected or not with SIV/17E-Fr at a multiplicity of infection of 0.1 were then exposed or not to 100 units of recombinant macaque IFNB1 per milliliter of culture medium, creating four experimental conditions. We compared the change from pre-infection at post-treatment time points of two, seven, 14, and 21 days, with the untreated, uninfected condition as the reference at each time point. At two days post-treatment, SAMHD1 levels were significantly upregulated in all interferon and infection conditions, including an approximately eight-fold upregulation in response to interferon (Fig. 5). Thereafter, SAMHD1 remained upregulated in the presence of exogenous interferon but not in the SIV alone condition. The large fold change of SAMHD1 in astrocytes compared with macrophages suggests, with the caveats noted above, that astrocytes may contribute a larger portion of the observed upregulation of SAMHD1 transcript in brain. Furthermore, it is interesting to note the comparatively greater response of SAMHD1 in the astrocyte, generally considered a non-permissive cell type, versus the permissive macrophage. However, since the astrocytes used in these *in vitro* experiments do support viral replication, additional work will be needed to elucidate the details of the role of SAMHD1 in enforcing viral latency in astrocytes *in vivo*.

## Discussion

We report that SAMHD1 expression is significantly upregulated in thalamus during retroviral infection and tracks with the Type I interferon response as represented by MX1. Our *in vitro* results suggest that, in part, this upregulation of SAMHD1 may be driven by the strong response of astrocyte SAMHD1 to Type I interferon—specifically IFNB1—as well as a comparatively minor and short-lived response to retrovirus exposure. We cannot rule out that this high level of SAMHD1 expression may help to protect astrocytes from retrovirus infection, even in the case of Vpx-expressing SIV. Although the contribution of macrophages is substantially less clear due to contradictory results in the literature, our findings suggest that macrophages may also contribute an augmented complement of SAMHD1 following IFNB1 exposure.

Despite the upregulation of SAMHD1 in thalamus during SIV infection, SAMHD1 transcript levels do not correlate negatively with viral load. Thus, the arms race against the retrovirus is lost overall, likely in part due to SIV counteraction of SAMHD1 protein in infected cells. This contrasts with our results on HIV-1 in the periphery, where, in a study of PBMC of HIV-1 elite suppressors and chronic progressors, a negative correlation of viral load with MX1 and SAMHD1 was observed in viremic individuals^31^. It will be interesting to follow up on these findings in HIV-infected brain.

Strategies to upregulate SAMHD1, for example during “shock and kill” reactivation therapy^50^, could supplement the actions of traditional antiviral drugs and further prevent the spread of any newly produced virus. Transcriptional regulation is of course not the only determinant of protein activity. One form of post-transcriptional regulation is microRNA-mediated repression: regulation of the SAMHD1 transcript may include post-transcriptional blocks such as miRNA-mediated degradation or translational attenuation. As we reported recently^43^ and at the 2013 Conference on Retroviruses and Opportunistic Infections^51^, miRNAs with seed sequence matches to SAMHD1 that appear to bind to the transcript include miRs-34a, -125b, -150, -155, and the -181 family. This effect has recently been confirmed for miR-181a and miR-155^42^ and may occur for other miRNAs as well^7^. The finding that host miRNAs contribute to SAMHD1 regulation could form the basis of a SAMHD1-enhancing strategy as mentioned above. Protein-level activity is also governed by post-transcriptional factors including phosphorylation state^52^,^53^ and multimerization^54^. Blocking Type I interferon signaling in myeloid and plasmacytoid dendritic cells allowed the cells to become permissive to HIV-1 infection^55^, and the antiviral form of SAMHD1, without phosphorylation on residue Threonine 592, is promoted by interferon^53^. Accordingly, the phosphorylation and multimerization states of SAMHD1 should also be investigated further in the CNS.

## Methods

### Animal studies and ethics statement

Studies of animal materials were conducted using archived samples from previous, concluded pigtailed macaque studies involving dual-inoculation with an immunosuppressive SIV swarm and a neurotropic clone^56^; no new animal subjects were involved in this project. Characteristics of the subjects are outlined in Table 1. In addition to uninfected controls, post-infection time points examined were 0, 4, 7, 10, 14, 21, 42, 56, and 84 dpi. All animal studies were approved by the Johns Hopkins University Institutional Animal Care and Use Committee and conducted in accordance with the Weatherall Report, the Guide for the Care and Use of Laboratory Animals, and the USDA Animal Welfare Act.

### Macrophage differentiation

Peripheral blood mononuclear cells (PBMCs) were isolated from de-identified blood products by standard Percoll protocol as described previously^57^,^58^. PBMCs were cultured at a density of 4 × 10^6^ cells per well in 12-well plates or 2 × 10^6^ cells per well in 24-well plates in macrophage differentiation medium (MDM) with 20% serum [MDM20: Dulbecco’s modified Eagle’s medium (DMEM, Invitrogen), 20% human serum (GemCell), 2 mM L-Glutamine (Sigma), 10 mM HEPES (Gibco), 10 mM sodium pyruvate (Sigma), 50 ng/ml M-CSF (R&D), and 2 mg/ml Gentamicin (Gibco)] for one week, with half-volume re-feeding at three days post-plating. Note that human serum lots were selected for minimal pyrogen activity and tested in cell culture before use. After seven days, supernatants were removed and adherent macrophages were washed with PBS. Medium was replaced with MDM with 10% human serum.

### Macrophage infection, harvesting and RNA isolation

Differentiated macrophages in 12-well plates were infected with HIV-1-BaL (50 ng p24) for 4–6 hours, and virus was removed with extensive washes with PBS. Infected and uninfected cells were harvested in mirVana RNA lysis buffer (Ambion/Life Technologies) day 0 and at 2, 7, 9, 14, and 21 dpi and stored at −80 °C. Supernatants were also collected and stored at −80 °C.

### Short-term macrophage cytokine treatment

Macrophages were sham treated or treated with 100 units/mL of recombinant human IFN-beta or 20 ng/mL TNF-alpha (PBL Interferon Source, now PBL Assay Science, Piscataway, NJ). Lysates were collected at 2, 4, 8, 12, and 24 hours post-treatment as described above.

### Astrocyte infection with SIV

Primary macaque astrocytes were purchased from Cambrex and cultured in astrocyte growth media with supplements (Lonza) as described previously^41^. Twenty-four hours prior to infection, astrocyte medium was changed to DMEM with 10% fetal bovine serum. Cells were infected at moi = 0.05 with SIV/17E-Fr^59^. At six hours post-infection, cells were washed five times with PBS and re-fed with fresh medium. The next day, cells were treated or not with 100 units/mL of recombinant macaque IFN-beta (PBL Assay Science, Piscataway, NJ). Cells were half re-fed twice weekly with medium containing the cytokine as appropriate. Astrocyte lysates at the specified time points were collected in mirVana lysis buffer and stored at −80 °C until RNA purification.

### RNA isolation from tissue and cell culture

Thalamus tissue from the rapid *Macaca nemestrina* model of HIV CNS disease was obtained after long-term maintenance at −80 C. RNA was extracted with a modified Trizol protocol with column clean-up as previously described^60^. 30–50 mg frozen tissue was placed into 1 mL Trizol (Invitrogen, Grand Island, NY) in 2 mL screw-cap tubes, and one tube of Lysing matrix D (MP Biomedicals, Solon, OH) was added. Tissue was disrupted for 2 × 30 s using a desktop bead beater with five minutes on ice interspersed. Tubes were spun to collect beads and homogenate, and 200 μl chloroform was added. Tubes were firmly capped and shaken vigorously for 1 min, then centrifuged at 12,000 × g in an Eppendorf C2415 centrifuge for 15 min at 4 °C. The aqueous phase was transferred to a new tube, with care taken not to disturb the interphase. 1.25 volumes 100% ethanol was added. Sample was applied to mirVana miRNA isolation kit columns for cleanup (Ambion/Life Technologies).

Total RNA was extracted from frozen cell culture samples using the mirVana miRNA isolation kit (Ambion/Life Technologies), following the manufacturer’s protocol. RNA for each experiment was isolated together to avoid batch effects. RNA concentration of all samples was assessed by NanoDrop, and integrity of astrocyte RNA and several additional samples was assessed by BioAnalyzer. All RNA was stored at −80 °C.

### Quantitative PCR

cDNA was generated using the High-Capacity cDNA Reverse Transcription kit (Ambion/Life Technologies, #4368814) and manufacturer’s protocol. Pre-designed TaqMan Assays (Applied Biosystems/Life Technologies) were used to quantitate SAMHD1, Mx1, GAPDH, and ACTB from human (Hs00210019_m1, Hs00895608_m1, Hs02758991_g1, and Hs99999903_m1, respectively) and macaque (Rh02869978_m1, Rh02842279_m1, Rh02621745_g1, and Rh03043379_gH, respectively). Amplification reactions were performed with the CFX96 optical system (BioRad). Delta-delta Cq-based normalization was to the average of housekeeping genes GAPDH and ACTB (Delta Cq) and the average of control, uninfected samples (Delta-delta Cq).

### Data analysis, statistical methods, and figures

Data processing and analysis were conducted using tools from Microsoft Excel, GraphPad Prism, and XLStat. Analysis of Variance (ANOVA) was used to calculate p values. Significance of correlations was calculated as the two-sided probability value of the Pearson correlation coefficient.

## Additional Information

**How to cite this article**: Buchanan, E. L. *et al.* SAMHD1 transcript upregulation during SIV infection of the central nervous system does not associate with reduced viral load. *Sci. Rep.* **6**, 22629; doi: 10.1038/srep22629 (2016).

## Figures and Tables

**Table 1:** Characteristics of Pigtail Macaques Used in Study.

Animal	D.P.I.	Sex	Age (years)	Brain Viral Load	Mane-A*10 Status	CNS Disease
1	4	M	0.7	781	NT	None
2	4	M	0.7	67	NT	None
3	4	M	1.1	94	NT	None
4	4	M	1.0	125	NT	None
5	4	M	1.0	146	NT	None
6	4	M	0.9	258	NT	None
7	7	M	3.3	1788	+	NT
8	7	M	3.6	2932	−	NT
9	7	M	3.3	6573	−	NT
10	7	M	3.0	6434	−	NT
11	7	M	3.5	39725	−	NT
12	7	M	2.3	1402	−	NT
13	10	M	2.0	2604	NT	Mild
14	10	M	1.9	92130	NT	Mild
15	10	M	1.7	6314	NT	Mild
16	10	M	1.2	67957	NT	Mild
17	10	M	1.9	6088	NT	Mild
18	10	M	0.9	2072	NT	None
19	14	M	4.2	13180	−	Mild
20	14	M	5.0	12069	−	None
21	14	M	4.1	4160	−	Mild
22	14	M	5.0	13987	−	None
23	14	M	5.5	12100	+	None
24	14	M	4.2	3897	−	Mild
25	21	M	3.3	1040267	−	Mild
26	21	M	6.7	154926	−	Mild
27	22	M	5.0	136689	+	None
28	22	M	4.7	26410	−	Mild
29	23	M	4.8	45630	−	Mild
30	23	M	6.7	68870	−	None
31	41	M	3.2	NT	−	Mild
32	42	M	3.2	NT	−	Mild
33	42	M	4	NT	−	Moderate
34	43	M	3.9	NT	−	None
35	43	M	4.2	NT	−	Severe
36	44	M	1.3	NT	−	None
37	44	M	3.4	NT	−	Severe
38	44	M	5.7	NT	+	None
39	45	M	2.5	NT	−	None
40	55	M	4.5	15900000	−	Severe
41	55	M	6.6	1905333	−	Severe
42	55	M	5.3	121300	−	Mild
43	55	M	4.4	5725000	−	Severe
44	56	M	8.5	11880000	−	Severe
45	56	M	4.8	16607	−	Mild
46	56	M	4.8	382467	−	Moderate
47	56	M	6.3	63483	−	Mild
48	57	M	6.5	8658333	−	Severe
49	66	M	3.2	34430567	−	Severe
50	85	M	Unknown	3523	+	Mild
51	85	M	2.8	136927	+	Severe
52	86	M	3.3	1950	+	Mild
C1	N/A	M	3.5	Negative	NT	None
C2	N/A	M	2.9	Negative	NT	None
C3	N/A	M	3.1	Negative	NT	None
C4	N/A	F	3.4	Negative	NT	None
C5	N/A	F	3.4	Negative	NT	None
C6	N/A	F	2.7	Negative	NT	None

**Table 2:** Interferon-sensitive response elements identified in the promoter region of SAMHD1.

Sequence	Location	Strand	Similarity
Homo Sapiens: GXP_1491445 (1–602)			
ISRE	423–443	+	0.820
ISRE	553–573	+	0.839
Macaca Mulatta: GXP_1663244 (1–602)			
ISRE	420–440	+	0.820
ISRE	553–573	+	0.828
